# Intraoperative Profiling of the Supracrestal Implant Complex Minimizes Peri-Implant Crestal Bone Remodeling: The Guided Bone Profiling Concept

**DOI:** 10.3390/jfb16030093

**Published:** 2025-03-08

**Authors:** Milan Stoilov, Joerg Winterhoff, Lea Stoilov, Anastasia Timoschenko, Helmut Stark, Florian Heuser, Michael Marder, Dominik Kraus, Norbert Enkling

**Affiliations:** 1 Department of Prosthodontics, Preclinical Education and Dental Materials Science, University Hospital Bonn, 53111 Bonn, Germany; stoilov@uni-bonn.de (M.S.); dominik.kraus@ukbonn.de (D.K.); 2 Department of Reconstructive Dentistry and Gerodontology, School of Dental Medicine, University of Bern, 3012 Bern, Switzerland; joerg.winterhoff@t-online.de

**Keywords:** bone remodeling, emergence profile, implant success, immediate implant, late implant, immediate restoration, immediate loading

## Abstract

(1) Background: Early-stage bone resorption following implant placement can significantly impact the long-term success of implants. This study evaluates whether a fully digitally planned implant position based on the E-point concept, along with guided profiling of the supracrestal complex, contributes to improved stability of peri-implant bone levels. (2) Methods: 29 implants were placed in 27 patients utilizing both immediate (Group 1; n = 19) and delayed placement (Group 2; n = 10) protocols. Implant position and emergence profile were preoperatively determined and consistently executed through guided surgery and CAD/CAM-fabricated restorations. Due to the subcrestal positioning of the implant, a corresponding bone profiler with a guide pin was used to shape the emergence profile and prevent the provisional restoration from impinging on the proximal bone. Provisional restorations were immediately placed to support the emergence profile. Bone level changes were documented radiographically over a two-year period. The first Bone-to-Implant Contact Level (∆ fBIC), change in highest approximal Bone Level (∆ haBL), and formation of an emergence profile width (WEP) were measured. (3) Results: All implants and restorations survived after two years, no significant change in first Bone-to-Implant Contact Level (∆ fBIC = 0 ± 0.02 mm), no change in highest approximal Bone Level (∆ haBL) of −0.23 mm ± 0.71 mm, and formation of an emergence profile width (WEP) averaging 0.18 ± 0.19 mm. (4) Conclusions: Despite the initial stress on the bone caused by bone profiling, guided implant placement and bone shaping, supported by an immediate provisional, have a positive effect on peri-implant bone stability.

## 1. Introduction

Implantology is a well-established and effective method for restoring single-tooth gaps as well as more complex prosthetic rehabilitations, including full-arch restorations. In recent years, immediate implant placement with immediate loading protocols has gained popularity, particularly in the esthetic zone. Gallucci et al. [1] define this as a “Type 1A” implant placement protocol, offering the potential for excellent esthetic outcomes and high implant survival rates [2]. Preserving both hard and soft tissue volume is crucial for achieving these outcomes. However, adequate implant positioning is essential, as it significantly impacts the overall success of the implant and the restoration [3]. Therefore, implants should be ideally positioned to support the emergence profile of the restoration, ensuring proper soft tissue form and symmetry [4], and esthetic success in the final restoration. This restorative-driven surgical approach offers additional advantages, including the preservation of bone dimensions, reduced periods of uncomfortable provisional restorations, and shortened treatment times [5,6,7].

The use of an immediate provisional restoration is essential for optimal emergence profile conditioning [8]. After implant placement, the contour of the provisional restoration preserves, modifies, and supports the soft tissue, thereby contributing to a natural emergence profile [9,10]. A favorable emergence profile is also crucial for the health of the peri-implant tissues, as it influences the effectiveness of oral hygiene [11,12], as a sufficient amount of keratinized gingiva is particularly important in preventing peri-implant inflammation [13].

Furthermore, the midfacial soft tissue level is considered one of the most important factors for the esthetic appearance [14].

However, achieving this first requires the correct vertical and horizontal relationship between the implant and the peri-implant soft tissue. Only from this starting point can the provisional crown be contoured appropriately. The literature indicates that implant mispositioning, most often in the buccal direction, can lead to soft tissue recession and consequently result in an esthetically compromised outcome [15]. Fürhauser et al. addressed this issue in their study by defining the ideal emergence point (E_IDEAL_ point) as a reference for the buccal implant position [16]. They describe this point as the midfacial zenith of an existing or planned implant crown, viewed frontally at the same level as the corresponding tooth. From this reference, the buccal shoulder of the implant should be positioned at least 2 mm palatally to achieve optimal esthetic results. 

As described, the contour of the provisional crown is important for the success of the implant-supported restoration, as an esthetic implant-supported restoration emerges through the surrounding tissues like a natural tooth [17,18]. Gomez-Meda et al. [19] relate to these findings by introducing the esthetic biological contour concept (EBC), which defines the different zones of the emergence profile and provides guidelines for contouring the provisional restoration.

Further considerations regarding the emergence profile consider the vertical position or depth of the implant shoulder. Guidelines for implant positioning recommend placing the immediate implant approximately 3–4 mm apical to the future crown margin/zenith in bone-level implants within the comfort zone [20]. To achieve a natural emergence profile and minimize the restoration angle, narrower implant platforms often require an even deeper vertical placement [21], particularly when using an implant system with a conical internal implant–abutment connection. The emergence angle is also positively influenced, as the sufficient distance between the implant shoulder and the restoration zenith allows the recommended angle of ≤30° to be maintained [22].

Above all, the digital workflow allows for precise pre-implantation planning based on overlaid CBCT and STL data while also enabling the creation of a customized provisional restoration [23,24]. By the time of the implantation procedure, a surgical guide and a ready-to-use provisional restoration are available. If the planned implant position is accurately transferred to the patient’s mouth, the provisional restoration can be placed immediately. However, this workflow requires exceptional precision at every step, demanding meticulous execution from the dentist and the dental technician as it is a technique-sensitive procedure [24].

Nevertheless, in Type 1A protocols, a common issue is that the proximal bone interferes with subcrestally positioned implants. When attempting to insert the screw-retained provisional, the restoration may rest on the bone, requiring modification of the surrounding bone. To address this, so-called Bone Profilers can be used, which gradually cut away the excess bone around the coronal aspect of the implant, allowing the restoration to fully seat. This approach enables controlled and targeted bone reduction with prosthetically driven bone contouring. By anticipating the bone remodeling process post-implantation, the authors suggest a bone-protective effect. However, this is a very invasive procedure that sacrifices bone in a sensitive area, especially when considering the literature showing that implants placed into a fresh extraction socket experience less than 1 mm of bone resorption after one year [25].

Therefore, the aim of the present study was to analyze bone remodeling and peri-implant bone loss in immediate implants with immediate restoration (Type 1A) within a fully digital workflow, using guided bone profilers for conditioning the supracrestal complex. A comparison was to be made with delayed implants, which were also immediately restored with a prefabricated provisional restoration (Type 4A). It was hypothesized (H_0_) that intraoperative profiling of the supracrestal implant complex using guided bone profilers does not significantly reduce peri-implant crestal bone remodeling or peri-implant bone loss in immediate implants with immediate restoration within a fully digital workflow.

## 2. Materials and Methods

This prospective clinical observational study was conducted at the Department of Prosthodontics, Preclinical Education, and Materials Science at the Bonn University Hospital. Ethical approval was obtained from the Ethics Committee of the University of Bonn prior to the start of the study (096/20). Additionally, the clinical study was registered in the German Clinical Trials Register (DRKS00022273). A total of 29 implants were placed in 27 patients (9 females, 18 males) with an average age of 49 ± 20 years. Of these, 19 implants were placed following an immediate protocol with immediate restoration (Type 1A), while 10 implants were placed in a delayed manner but also immediately restored (Type 4A). Based on our power analysis, a total of 13 patients in Group 1 and 9 patients in Group 2 were required to achieve a statistical power of 0.8. This was based on an expected standard deviation of ± 0.37 mm for ∆ haBL (immediate implants with >12 months follow-up) [26] and aimed to detect a minimum difference of 0.5 mm in ∆ haBL after the healing period. The aim of the study was to investigate the effects of intraoperative guided shaping of the supracrestal complex on bone remodeling. This approach combined implant positioning, bone contouring, and the simultaneous placement of an immediate provisional restoration with an optimized emergence profile. Standardized periapical radiographs were used over a two-year period to monitor the peri-implant crestal bone level and emergence profile.

### 2.1. Inclusion Criteria

Patients were invited to participate in the study based on the following criteria:

Indication for a single-tooth implant in the anterior or premolar region;

Indication for immediate implantation (post-extraction) and immediate restoration;

Indication for late implantation with immediate restoration;

Inflammation-free, color-matched mucosa;

No gingival recessions, ideally with excess soft tissue;

Presence of interdental papillae;

Attached gingiva ≥ 2 mm;

Harmonious gingival contour profile;

Adequate bone volume to ensure primary implant stability ≥ 25 Ncm;

Approximately 3 mm of bone apical to the root tip to ensure primary stability;

Presence of a buccal alveolar wall;

No contraindications according to implant therapy guidelines.

### 2.2. Treatment Group Classification

Patients were divided into two main groups according to Gallucci et al. [1] and the ITI Treatment Guidelines [20]:

Group 1: Type 1A—immediate implantation with immediate restoration

The non-preservable tooth was extracted, the implant was placed immediately, and a prefabricated provisional crown was loaded immediately (Type 1A; 19 implants). Final crowns were inserted six months after implantation. The treatment followed the recommendations of Morton et al. [2] for Type 1A.

Group 2: Type 4A—delayed implantation with immediate restoration

The non-preservable tooth was extracted, and the implant was placed either after a necessary healing period due to individual bone conditions or into an already healed site. A provisional prosthetic restoration was placed immediately after implantation (Type 4A; 10 implants). Final crowns were inserted after six months.

In both groups, insertion depth, bone contouring, and design of the immediate provisional restoration were exclusively digitally planned and executed to optimize the final shaping of the supracrestal complex.

### 2.3. Implant Planning and Fabrication of Surgical Guide and Provisional Restoration

Following patient anamnesis, a CBCT scan (Promax^®^, Planmeca^©^, Helsinki, Finland) and an intraoral baseline scan (Carestream 3600, Carestream Dental^©^, Rochester, NY, USA) were performed for all patients. The data were exported in DICOM and STL formats and imported into the SMOP implant planning software (Version 2.15.4, Swissmeda AG^©^, Baar, Switzerland), where they were manually aligned using defined reference points. This overlay served as the foundation for virtual implant planning and positioning. Additionally, virtual wax-ups of the future crowns were created. To enable precise planning, digital wax-ups with the ideally planned emergence profile were incorporated into the process by overlaying them with the CBCT scan and the virtual study model within the planning software (Version 2.15.4). For Type 1A cases (Group 1), where the crown and emergence profile of the tooth to be extracted remained intact, the study model, including the existing tooth, was used as a reference for the virtual wax-up. In contrast, for Type 4A cases (Group 2), or when the crown was fractured in Type 1A cases, the virtual wax-up had to be created without reference to the original tooth.

The virtual implant planning and positioning were subsequently carried out by an experienced oral surgeon, who also performed all implant surgeries in this study. The surgeon is highly skilled in using SMOP software (Version 2.15.4) and has extensive experience in digital implant planning. The planning process adhered to the currently accepted criteria for implant placement [27] and the requirement to align the implant with the planned restoration [28]. In addition, the following specific requirements for implant positioning were implemented in this study:

- The implant position was planned and implemented in accordance with the ideal emergence point as described by Fürhauser et al. [16]. The vestibular implant shoulder was positioned 2–3 mm palatally and apically to the referencing emergence point. In this case, the emergence point was either the still intact highest point of the vestibular marginal gingiva of the tooth to be extracted or the corresponding contralateral tooth.
- The insertion depth was planned so that the implant platform was positioned 1–2 mm subcrestal to the lowest point of the vestibular bone lamella. The combination of subcrestal implant placement and the implant system used, with a reduced-diameter conical emergence (SIC Vantage Tapered, SIC Invent^©^, Basel, Switzerland), allowed for a harmoniously widening emergence geometry adapted to the natural tooth model, ensuring a favorable tissue excess.
- The implant diameter and length were selected to ensure that at least 3 mm of remaining bone was present apically. Oro-vestibularly, the implant should be positioned at least 2 mm palatally to the buccal lamella.
- Additionally, a steep implant angle was chosen to ensure proper screwing of the provisional or final crown.

Upon completion of the implant planning, the drilling guide was manufactured from Luxa Print Ortho Plus (DMG, Hamburg, Germany) using the 3D printer Prime 150 (MiiCraft^©^, Qingdao, Shandong, China). After finishing the post-processing of the drilling guide, it was inspected for fitting inaccuracies, and a marker was placed at the site of the drilling hole. This marker served to indicate the orientation of the implant index to the implanting surgeon, allowing for adequate fitting of the provisional restoration later.

In the next step, the implant planning data were exported in STL format and imported into the TRIOS^®^ Design Studio software (Version 2021.1, 3Shape^©^, Copenhagen, Denmark) to design the provisional restoration. This restoration was intended both to act as a wound closure and to shape the emergence profile. To ensure the proper shaping of the emergence profile and the supracrestal complex, the following considerations had to be considered during the design of the provisional restoration:

- As previously described in the virtual implant positioning, the emergence profile of the implant crown should match that of the original tooth or the corresponding contralateral tooth [16].
- An emergence angle (EA) of 30° should not be exceeded in the supracrestal complex area [22].
- Due to the palatally oriented position of the implant, a protruding (convex) curvature had to be applied vestibularly to esthetically compensate for the palatal position, ensure a tight seal with the tissue, and integrate the gap-filling bone substitute material [19].
- A titanium bonding base was used constantly with a neck height of 1 mm for both the provisional and final restoration.
- The provisional crown should have no occlusal or proximal contact with the antagonistic or adjacent teeth. A distance of at least 0.5 mm proximally and 1 mm occlusally should be maintained. There should also be no contact during dynamic excursion movements.
- The provisional restoration’s screw retention should be checked and ensured.

After the design of the provisional restoration was completed, it was milled from polymethyl methacrylate (PMMA Monocolor Disk, Yamahachi Dental^©^, Gamagori, Japan) using the CAM process (M4, Zirkonzahn^®^, Gais, Italy). The restoration was then polished, and after verifying the fit on the printed model, it was bonded with the appropriate titanium bonding base (SICvantage CAD/CAM Abutment, SIC Invent^©^, Basel, Switzerland) using Multilink Hybrid Abutment and Monobond Plus (Ivoclar Vivadent^©^, Schaan, Liechtenstein).

### 2.4. Fully Guided Implant Placement and Integration of the Provisional Restoration

**Figure 1:**
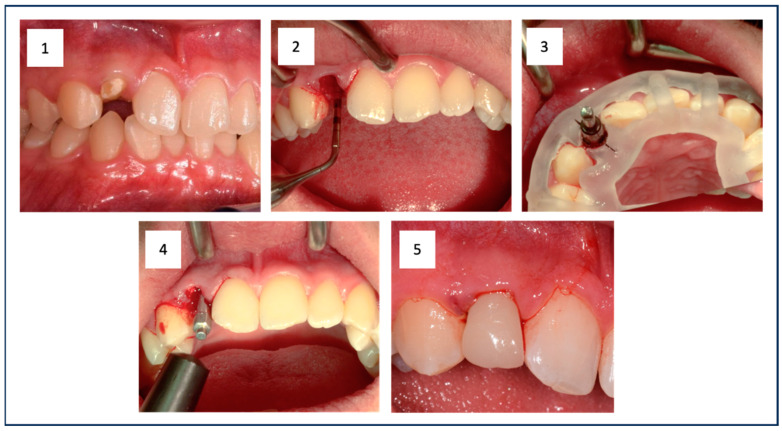
The initial situation shows the root remnant of the upper right lateral incisor (1). The empty extraction socket is visible after minimally invasive tooth removal (2). The situation after guided implant placement is shown, clearly illustrating the surgical guide and the properly oriented implant (3). This was followed by RFA measurement to document sufficient primary stability in addition to measuring the insertion torque (IT) (4). Finally, the prefabricated provisional restoration was fixed (5).

At the beginning of the implant surgery, the patients received local anesthesia, and if possible, the fit of the surgical guide was checked in the patient’s mouth. For patients in Group 1, atraumatic extraction of the tooth was performed, followed by careful curettage of the extraction socket (Figure 1(1,2)). For the patients in Group 2, a full mucosal-periosteal flap was raised, and the bone was exposed.

In both groups, the implant bed preparation was carried out using the SIC-Guided Surgery Kit (SIC Invent^©^, Basel, Switzerland). The preparation followed the manufacturer’s instructions and was based on the implant type, performed fully guided using the prepared surgical guide and cooled with sodium chloride solution (0.9%) (Figure 1(3)). A tapered implant with a conical internal connection (SIC vantage tapered, SIC Invent^©^, Basel, Switzerland) was then inserted. For the preparation and implant insertion, the Implantmed Plus SI-1023 drive unit with automatic torque control (W&H^©^, Bad Reichenhall, Germany) was used. This allowed the measurement of the insertion torque (IT) of the implant during insertion. Additionally, the ISQ value was measured with resonance frequency analysis (RFA) (Osstell^®^ IDX, Osstell AB, Gothenburg, Sweden) (Figure 1(4)). Cut-off values for the primary stability of the implant were set at an IT of ≥25 Ncm and an ISQ of ≥60. Adhering to or exceeding these values was essential to ensure sufficient primary stability for secure osseointegration in immediate restoration. Before the provisional restoration could be placed (Figure 1(5)), the jumping distance between the implant and the buccal bone lamella was filled with bone substitute (Cerabone^®^, Botiss Biomaterials, Zossen, Germany) for the patients in Group 1. For patients in Group 2, this step was not necessary.

**Figure 2:**
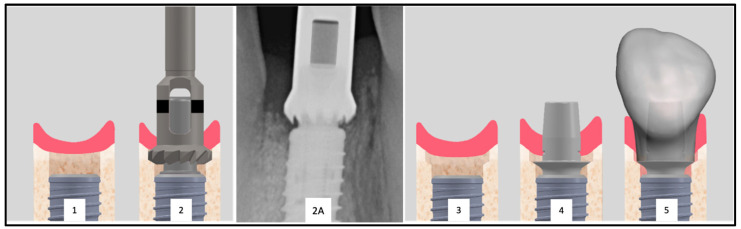
Inserted implant with clearly visible proximal bone overhangs (1). To allow the placement of the provisional restoration a bone profiler is used (2). Radiographic image of an exemplary Bone Profiler (2A). This tool reduces the bone of the supracrestal complex according to the diameter of the abutment (2). The conditioned bone is visible (3). The conditioning of the bone enables the seamless placement of the abutment (4) and the restoration (5).

Furthermore, the subcrestal placement of the implant (1.5 mm) led to proximal bone overhangs, requiring adjustment of the supracrestal complex with a bone profiler matching the implant and abutment size. This also enabled the optimized shaping of the emergence profile after implant insertion by contouring the crestal bone with the Bone Profiler System (SIC Invent^©^, Basel, Switzerland) (Figure 2). This system is specifically designed to shape the bone above the implant shoulder to match the desired emergence profile, with the profiler’s milling angle corresponding to that of the abutment (30°). The Bone Profiler shown in Figure 1 is a bone trephine drill used with a surgical contra-angle handpiece operating at approximately 50 rpm. It includes a mounted guide pin for precise control. The system consists of trephine drills in various diameters (4.0, 4.5, 5.0, and 6.0 mm), corresponding to the implant and abutment diameters, allowing for the shaping of the planned ideal emergence profile in the crestal bone. To prevent damage to the implant shoulder, the guide pin is securely fixed to the implant, directing the Bone Profiler and limiting its depth to 0.02 mm above the implant shoulder.

In Group 2 (Type 4A), the Bone Profiling System was not used, as no bony overhangs were present, allowing for the seamless insertion of the provisional restoration.

The Bone Profiler with a circular diameter of 4.5 mm was used for the titanium bases with a diameter of 4.05 mm at the crown-abutment junction (CAJ), and the profiler with a preparation diameter of 5 mm was used for titanium bases with a CAJ diameter of 4.5 mm. This ensured a lateral clearance of 0.2–0.25 mm for the base of the emerging crown (Figure 2).

After fitting the provisional crown and ensuring proper fit, it was screwed in with a torque of 20 Ncm. The screw access channel was temporarily sealed with sterile Teflon tape and a temporary resin (Clip, Voco^©^, Cuxhaven, Germany) (light curing for 40 s). In Group 2, sutures were then placed (Prolene 5-0, Ethicon^©^, Raritan, NJ, USA). Finally, any static and dynamic occlusal contacts, as well as any existing proximal contacts, were eliminated to prevent implant movement. After the implant surgery, a standardized periapical X-ray was taken for all patients.

After six months, provisional restorations were replaced with completely digitally planned and milled zirconia crowns (Prettau 2 Dispersive, Zirkonzahn^®^, Gais, Italy) on titanium bonding bases (SICvantage CAD/CAM Abutment, SIC Invent^©^, Basel, Switzerland). This involved an intraoral scan (Carestream 3600, Carestream, Rochester, NY, USA) using a scan body (SIC vantage Scan Adapter, SIC Invent^©^, Basel, Switzerland). The crown design was once again performed using the TRIOS^®^ Design Studio Software (Version 2021.1, 3Shape^©^, Copenhagen, Denmark). The crowns were milled using the M4 unit (Zirkonzahn^®^, Gais, Italy) and bonded with Multilink Hybrid Abutment and Monobond Plus (Ivoclar Vivadent^©^, Schaan, Liechtenstein). After fitting, the restorations were screwed in with a torque of 20 Ncm, and the screw access channel was sealed with sterile Teflon tape and a definitive composite filling material (Tetric Evo Ceram, Ivoclar Vivadent^©^, Schaan, Liechtenstein).

### 2.5. Assessment of Vertical Bone Changes

**Figure 3:**
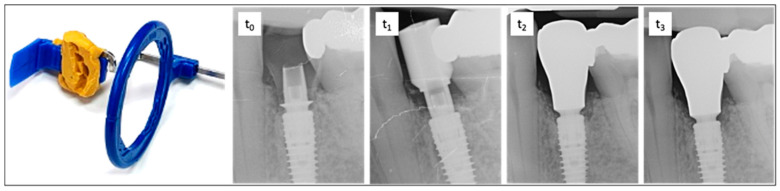
On the left is the radiographic film holder, which was customized with malleable silicone to ensure a reproducible position for all imaging sessions. On the right is an exemplary sequence of dental radiographs taken at time points t_0_, t_1,_ t_2_, and t_3_. t_0_—time of implantation. The subcrestal position of the implant and bonding base is clearly visible. Adaptation using the Bone Profiler System enabled the insertion of the provisional restoration by removing obstructive proximal bony overhangs and shaping the emergence profile. t_1_—six months after implant placement with a scan body. t_2_—one-year follow-up showing bone remodeling and the final restoration. t_3_—two-year follow-up demonstrating stable bone conditions around the implant and completed remodeling.

To assess the vertical bone changes, intraoral radiographs were taken at the following time points: implantation (t_0_), six months postoperatively or at the placement of the final crown (t_1_), one-year follow-up (t_2_), and two-year follow-up (t_3_). These radiographs were obtained using the right-angle technique with digital storage phosphor plates (VistaScan Image Plate Plus and VistaScan Perio, Dürr Dental, Bietigheim-Bissingen, Germany; ProX, Planmeca^©^, Helsinki, Finland). A Rinn film holder was used, which was individualized for each patient with an impression silicone (Panasil^®^ Putty, Kettenbach^©^, Eschenburg, Germany) to ensure the comparability of the radiographs in terms of position, angle, and the distances to be measured (Figure 3).

The radiographs were evaluated by two calibrated examiners using the software “IC Measure 2.0” (version number: 2.0.0., The Imaging Source Europe^©^, Bremen, Germany). Through an integrated conversion process with a calibration tool, defined measurements in millimeters (to two decimal places) were derived from the pixel values. Although the second decimal place (range 1/100 mm) was not clinically distinguishable, it was retained for trend representation and average value calculations. Given the average deviations of ± 0.02 mm, as determined by the calibrating individual (measuring threaded sections with known dimensions), rounding to one decimal place would have resulted in greater inaccuracy.

**Figure 4:**
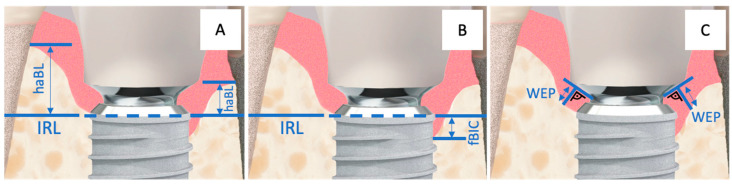
Implant–abutment junction with the corresponding measurement points: (**A**) = haBL, (**B**) = fBIC, and (**C**) = WEP. The horizontal, partially dashed line represents the Implant Reference Level (IRL).

For all evaluations related to the vertical bone–implant contact level, the crestal target height of osseointegration at the implant was defined as the reference (Implant Reference Level = IRL) (Figure 4A,B). The IRL is the boundary line on the implant where the surface conditioning (roughness, sandblasted-etched) of the implant meets the machined phase of the implant shoulder.

The highest apical bone level (haBL values) and the first bone-to-implant contact (fBIC values) were referenced to the IRL (Figure 4A,B). Additionally, the width of the hard and soft tissue emergence profile (WEP) around the provisional and later the final restoration was measured by determining the distance from the abutment–crown junction (ACJ) on a reference line (best fit next to the base of the abutment) to the bone of the emergence profile at a right angle (Figure 4C).

Since the measurements primarily concerned vertical distances, calibration in the vertical direction was essential for realistic results. Additionally, when using image plate technology for intraoral radiographs, the risk of unintended bending in the vertical direction due to the limiting anatomical structures of the palate and floor of the mouth should not be overlooked. For calibration in the vertical orientation, identifiable structures on the implant with known distances were used as a reference. In this case, the distance between five thread peaks corresponds to a real distance of 3 mm (implant-diameter 3.0 and 3.7) or 4 mm (implant-diameter 4.2 and 4.7), depending on the implant used.

**Figure 5:**
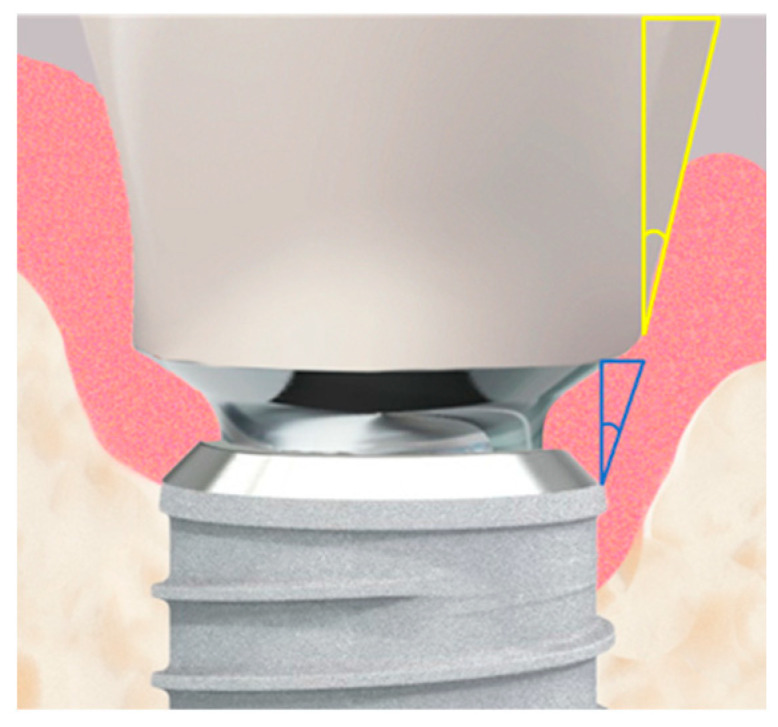
The implant–abutment junction is shown in high magnification. Two measured angles are illustrated: the actual emergence angle of the restoration is shown in yellow, while the fixed angle between the implant shoulder and the abutment, shown in blue, is 10°.

The emergence angles (EA) of the restorations were assessed by creating a “best-fit” line mesially and distally along the outer crown contour. This line was defined from the point where the crown begins from the titanium base (Crown-Abutment Junction, CAJ) to a point 5 mm (vertically) above the implant platform level. The angle of this line to the vertical axis of the implant was measured and documented (Figure 5).

### 2.6. Evaluation and Analysis of the Measurement Series

The change in bone height at the implant (∆ first Bone-to-Implant Contact, fBIC) was compared with the baseline situation at t0. It was calculated from the fBIC values at t_0_ and subsequent time points t_1_ to t_3_ (∆ fBICt_(x)_ = fBICt_(0)_ − fBICt_(y)_). For all implants where the Implant Reference Level (IRL) was completely covered by bone, the fBIC was defined as a nominal zero value, i.e., 0 mm. For implants where the fBIC mesially or distally fell below the IRL in an apical-coronal direction, the corresponding distances to the IRL were recorded with negative values. Bone heights that visually exceeded the implant–abutment junction (IAJ) level were not measured in the fBIC analysis; these bone areas were instead considered in the additional haBL and WEP analyses.

The development of the subpapillary bone height in the supracrestal complex was evaluated by measuring the highest apical bone level (haBL) in the mesial and distal proximal spaces in comparison to the IRL at t0 and subsequent time points (t_1_ to t_3_) (∆ haBLt_(x)_ = haBLt_(0)_ − haBLt_(y)_). Values for bone loss were reported with a negative sign.

Additionally, the width of the emergence profile (WEP) was measured mesially and distally between the crown-abutment junction (CAJ) and the bone of the emergence profile at a right angle. This measurement was compared for each observation period to the value at the time of implantation (t_0_) (∆ WEPt_(x)_ = WEPt_(0)_ − WEPt_(y)_). Decreases in width were represented with a negative sign.

The emergence angle (EA) of the prosthetic restoration (Figure 5) was measured at t_1_ during the insertion of the restoration against the longitudinal axis of the implant and documented in degrees.

### 2.7. Statistical Analysis

Due to the small sample size (n = 29 implants), the data from the mesial and distal aspects of each implant were summarized at the implant level to reduce the complexity of the statistical models. Unless otherwise specified, the analyses were conducted at the implant level. The primary outcome variables were WEP, haBL, and fBIC, alongside implant and crown survival. Due to the limited value ranges of WEP, ∆ WEP, and fBIC, logarithmic values or log differences were used for statistical analyses. All tests were performed at a significance level of α = 0.05. 95% confidence intervals are indicated in [square brackets].

For the analysis of haBL, a Repeated Measures Mixed Model was used, where patients and implants were defined as subject variables and time as the within-subjects variable. Predictors and their interactions with time were included as fixed factors to model changes in haBL over time.

WEP values were log-transformed, and separate mixed model analyses were performed for t_2_ and t_3_ to identify significant influences on the overall WEP after bone remodeling. For modeling ∆ WEP, log differences (logWEP[t_3_]–logWEP[t_0_]) were used as the dependent variable.

In the models, the patient’s gender, age (in years), follow-up time (in months), implant position (molars versus anterior teeth), implant protocol (Type 1A versus Type 4A), and emergence angle (°) were considered as predictors. Where applicable, logWEP[t_0_], fBIC[t_0_], and haBL[t_0_] were also included as predictors. Effect estimates were calculated using the Satterthwaite method for unbalanced data. For multiple comparisons, the sequential Bonferroni correction was applied. Unless otherwise stated, values are presented as estimated means [95% confidence intervals].

For the analysis of the fBIC distribution at the different time points, a Related Samples Friedman Test was performed.

The statistical analyses were conducted using IBM SPSS 27.0 software (IBM, Armonk, NY, USA). The study was reported in accordance with the STROBE guidelines, accessible through the EQUATOR network. 

To reduce the complexity of the statistical models, the analyses were generally performed and evaluated at the implant level. In cases where data quality allowed for analysis at the aspect level (mesial and distal implant aspects), these values were used.

## 3. Results

In the present study, 29 implants were placed in 27 patients. Of these, 19 implants were classified as Type 1A (Group 1), and 10 implants as Type 4A (Group 2). The patients had a mean age of 49 ± 20 years, with 9 female and 18 male participants. Follow-up appointments were conducted at 7 ± 4 months (t_1_), 13 ± 1 months (t_2_), and 24 ± 0 months (t_3_). All placed implants and definitive restorations remained intact and functional after 24 months. No complications or failures were observed, resulting in a survival rate of 100%. For clarity, additional patient and treatment characteristics of the study group are summarized in Table 1.

### 3.1. Development of the Bone Level in the Approximal Area (∆ haBL) 

Overall, the highest bone level in the proximal neighboring spaces to the adjacent tooth (haBL; aggregated at implant level) did not change to a significant or practically relevant extent (−0.259 mm [−0.543 mm to 0.024 mm]; p = 0.071) over the two-year follow-up period. Although haBL at implants in Group 2 (Type 4A) was insignificantly but consistently higher than implants in Group 1 (Type 1A) over all time points (*p* = 0.667; Table 2), no significant effect of immediate versus late placement on ∆ haBL over time has been detected (*p* = 0.802; Table 2).

The mixed effects model revealed no significant influence of any predictor on the change in proximal bone height (Δ haBL). Interestingly, however, patient sex seems to determine the ‘absolute’ height of haBL, as haBL is, on average, 1.1 mm [0.1 mm to 2.2 mm] lower in females than in males (*p* = 0.034). This absolute effect, however, does not necessarily reflect the actual proximal bone height but might merely reproduce the implant insertion depth, as haBL is calculated relative to the Implant Reference Level. The difference in haBL between the genders was consistent across all time points, so for Δ haBL, i.e., remodeling effects, no significant differences were discovered.

### 3.2. First Bone-to-Implant Contact (fBIC)

Vertical bone coverage of the implant surface intended for osseointegration (apex to IRL) was measured via the first Bone-to-Implant Contact in relation to the IRL (fBIC). The fBIC was recorded from t_0_ to t_3_ for mesial and distal aspects and aggregated at the implant level (Table 3).

There were only a few cases with incomplete vertical bone coverage of the implant surface (fBIC < 0) at any given time point. Because of the heavily skewed and bounded nature of the data in conjunction with the small number of implants without complete vertical bone coverage, no parametrical model could be fit. Related Samples Friedman Test revealed no significant differences in the distribution of fBIC between the time points (*p* = 0.146). Overall, considerable stability of the fBIC level was detected over the observation period. At the end of follow-up, only three implants exhibited minimal reduction in fBIC (Group 1 (Type 1A): two implants; Group 2 (Type 4A): one implant).

### 3.3. Evolution Emergence Profile Width (WEP)

**Figure 6:**
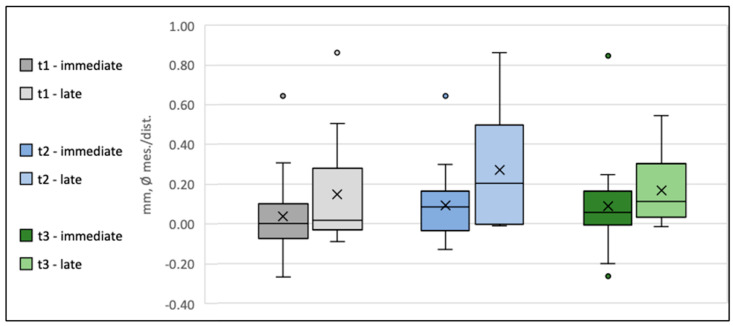
Box plot diagram of the development of the width of the emergence profile (Δ WEP) at time points t_1_, t_2_, and t_3_ for Group 1 (Type 1A) and Group 2 (Type 4A). The crosses indicate the mean values, the horizontal lines within the boxes represent the medians, whiskers denote the minimum and maximum measured values, and the circles indicate the respective outliers.

WEP indicates the clearance between the reference level (crown abutment junction (CAJ)) and the bone of the emergence profile. In cases when the abutment base touched the bone, WEP was recorded as 0, as was frequently the case at the time of insertion. Thus, at t0, the overall WEP (0.06 mm [0.02 mm to 0.09 mm]) was relatively small and subsequently increased to 0.19 mm [0.11mm to 0.26mm] at the end of the follow-up (t_3_), with the most prominent change in WEP occurring between the six months- and the one-year follow-up period (Table 4 and Figure 6).

Because of the bounded nature of WEP at t_0_ und t_1_, a mixed model of the WEP log-differences between the follow-up time points (t_x_) and t_0_ was used (logWEP[t_x_]–logWEP[t_0_]) to detect potential predictors on WEP change. To evaluate whether differences in initial WEP[t_0_] impact the remodeling activity during follow-up, WEP[t_0_] was included as a predictor in the model. In this regard, a significant influence of the initial WEP[t_0_] on ΔWEP[t_3_–t_0_] was detected (*p* = 0.000). Every 1% increase in WEP[t_0_] resulted in a reduction in ΔWEP[t_3_–t_0_] by 0.84% [1.121–0.559%] (*p* = 0.000), indicating that higher WEP values at provisional placement will tend to level out over the follow-up period. Accordingly, ANOVA of logWEP at one-year and two-year follow-ups revealed no detectable influence of any predictor on absolute WEP after the remodeling phase (*p* = 0.255).

### 3.4. Emergence Angle (EA) of Inserted Crowns

All definitive restorations, regardless of group affiliation, exhibited an emergence angle of ≤30°. The smallest emergence angle was 1.6°, and the largest was 29.8°. No correlation could be established between the emergence angle and the implantation protocol, or the implant location or tooth morphology, respectively.

## 4. Discussion

The use of dental implants has proven successful over the past decades, with reported survival rates in the literature ranging from 82% to 96% [29,30]. The survival rates for prosthetic restorations are also reliably high, with values between 78% and 95% [31,32]. However, in the anterior region, mere implant osseointegration and prosthetic survival alone are not sufficient to achieve a satisfactory outcome. Esthetics play a determining role in the success of an implant-supported crown in the anterior zone.

Several factors influence the final esthetic result, including adequate soft and hard tissue availability [33,34], implant positioning [3], and the formation of the emergence profile using a properly designed provisional restoration [10,35]. The introduction of digital techniques in implantology and prosthetics has enabled detailed analysis of anatomical structures, allowing for precise implant planning and emergence profile design. This enhances predictability and provides clinicians with greater security in achieving esthetically pleasing outcomes. In this context, immediate implant placement with immediate restoration is gaining increasing attention. This protocol, classified as Type 1A, also demonstrates high survival rates (98.4% with a mean follow-up of 28.9 months (SD = 15.2; range 12–60 months) [1] and aligns with the concept of preserving the existing soft and hard tissue anatomy to optimize esthetic results [9,10]. In the present study, we were able to demonstrate a 100% survival rate for immediate implantation and immediate loading after two years. This aligns with the very favorable survival rates described in the literature for Type 1A procedures today.

The stringent inclusion criteria employed in our study may be a key factor contributing to the 100% implant survival rate observed. These criteria are critical for minimizing confounding variables and ensuring a homogenous study population, which is essential for the validity and reliability of results in a prospective clinical study [36]. However, it is important to recognize that such restrictive criteria may limit the external validity and generalizability of our findings to broader clinical practice, where a more diverse patient population is typically encountered. While this methodological approach ensures high internal validity, it may not fully reflect the challenges and outcomes encountered in routine clinical settings. 

The present study specifically focused on the stability of the supracrestal complex and the shaping of both the soft and hard tissue emergence profile. Following ideal implant placement, the initial conditioning of the supracrestal complex was achieved using a guided bone profiler, followed by shaping and support with a digitally preplanned and preoperatively fabricated provisional restoration. The bone dimensions at immediate implant sites typically show a reduction of approximately 0.5–1.0 mm in both vertical and horizontal aspects 4–12 months following surgery [37].

In our study, we assessed peri-implant bone remodeling using three different parameters (fBIC, haBL, and WEP). Over the two-year study period, there were minimal changes in the values, with the greatest variation observed in the WEP values, which showed an overall change of 0.19 mm (range: 0.11 to 0.26 mm).

This is consistent with values reported in the recent systematic review by Dioguardi et al. [38]. This review found a range of marginal bone loss from 0.32 mm after one year [39] to 1.9 mm after up to 3 years [40]. Similarly, Bernard et al. [41] assessed 302 implants over a period of up to 3 years, reporting a marginal bone loss of 0.7 ± 1.3 mm in the guided surgery group and 0.5 ± 0.6 mm in the control group. Åkesson et al. [42] found a mean marginal bone level change of 0.6 mm for guided surgery cases, with no significant difference between reconstructed and non-reconstructed sites. These findings underline the variability in bone remodeling outcomes, which in turn supports the need for further investigation into factors such as socket buccal wall thickness and their relationship with dimensional changes in the bony ridge.

Although this study, with a relatively small number of subjects, has limited statistical power in some aspects, it can provide valuable insights into a previously underexplored topic: the intraoperative aspects of shaping the supracrestal complex according to the procedure described in relation to short- and long-term peri-implant bone preservation. The availability of standardized radiographs for all follow-up periods proved advantageous for evaluation. Even though they represent a three-dimensional situation in only two dimensions and, therefore, have a certain degree of inaccuracy, they still appear to provide reliable trends in bone remodeling within the measurable areas. 

The preservation of peri-implant bone stability has been one of the highest priorities in implantology since its inception. In this context, immediate implantation and immediate restoration have increasingly come into focus. Numerous publications and conferences have addressed biological, morphological, and functional parameters in the healing, remodeling, and osseointegration process, as well as the growing possibilities of digital planning and workflows [43,44,45,46]. A key aspect is whether and to what extent the initial formation phase of the supracrestal complex can positively influence long-term hard and soft tissue stability as well as esthetics. The interplay between implant positioning, restorative parameters, and surrounding tissue structures plays a crucial role in this process. In particular, the effects of the initial bone shaping and the design of immediate prosthetic structures, which influence the emergence profile, should be highlighted. In addition to morphological aspects, biomechanical factors must also be considered.

The initial emergence profile is formed during a phase of increased tissue metabolic activity, such as through extractions and bone preparation. Additionally, static and dynamic stimuli—such as loading and micromovements—can influence the resorption and formation phases of hard and soft tissues [47]. It has been shown that immediate loading, once appropriate primary stability is achieved, not only leads to good osseointegration but also contributes to the regeneration of the surrounding bone, resulting in higher bone volume and bone quality compared to non-loading protocols [48,49]. From this perspective, a one-stage procedure, where the bone alignment is oriented towards the implant from the outset, seems preferable to the conventional two-stage procedure. The latter involves an initial arbitrary bone formation to close the socket, followed by a later realignment towards the implant. A prospective approach to bone guidance from the very beginning not only ensures direct attachment of the bone to the implant surface but also allows for the planned shaping of the supracrestal complex through the temporary crown.

The shaping of the emergence profile by the crown plays a crucial role in maintaining both hard and soft tissue stability and later helps prevent bone loss associated with peri-implantitis [19]. Various studies thus focus on the design of the implant crown or provisional restoration to shape an ideal emergence profile that supports the long-term preservation of hard and soft tissues [19,50,51].

Based on the ideal emergence point described by Fürhauser et al. [16], this study designed the emergence profile of both the provisional and definitive crowns according to the EBC concept [19] while maintaining an emergence angle of less than ≤ 30° [52]. This approach resulted in stable peri-implant soft and hard tissue conditions throughout the entire two-year study period, which may also reflect the benefits of the appropriate emergence profile design. 

To shape the ideal emergence profile and accommodate the subcrestal positioning of the implant, conditioning of the supracrestal complex is essential (removal of bony overhangs). In the present study, this was achieved using the Guided Bone Profiling System. To our knowledge, no study currently addresses the specific influence of invasive shaping of the supracrestal bone combined with immediate implant placement and immediate loading on the long-term stability of the peri-implant bone. However, the Surgical Crown Lengthening Concept (SCL), designed to re-establish biological width and extend the supragingival tooth structure for restorative or aesthetic purposes, provides a relevant analogy. In a study by Guarnieri [53], stable periodontal tissue conditions were observed after 15 years. The bone level was reduced during surgery based on the future prosthetic margin and the predetermined biological width. This approach is comparable to the method used in the present study, where the ideal emergence profile was determined through correct implant positioning and invasive bone shaping using a bone profiler. The results of our study also demonstrate stable soft and hard tissue conditions after two years.

Guiding the ‘final’ shape of the emergence profile from the outset helps minimize iatrogenic tissue remodeling during the prosthetic restoration phases. A similar philosophy, focusing on the supra-implant soft tissue collar as part of the supracrestal complex, is embodied in the “One Abutment One Time” concept [54], which aims to improve tissue preservation [55]. However, this concept has a fundamental disadvantage: soft tissue changes during the prosthetic phases (temporary/definitive crown) may require intraoral corrections, such as tissue-affecting grinding procedures. The approach of using an immediate temporary restoration with a digitally planned and implemented emergence profile, followed by replacement with the final crown after six months using the same congruent emergence profile, represents a logical evolution in treatment methodology. While initially carried out with analog methods, digital techniques now offer more precise and consistent implementation [56].

This advancement avoids further irritation of peri-implant hard and soft tissues caused by reworking and frequent changes to the emergence profile, which could lead to new inflammatory and resorptive reactions. This is especially evident when comparing it to the conventional method of first shaping the gingiva with a gingiva former and then inserting an abutment crown. Gingiva formers typically have cylindrical or widening geometries and are circular when viewed from above. However, the morphological variation of natural teeth in a comparable vertical position is much greater. For example, in the upper incisor region, asymmetric oval geometries are common. Therefore, the goal is to optimize the emergence profile early on, considering the ideal position of the E_IDEAL_ point so that the emerging crown mimics the natural tooth [16].

By anticipating the ideal emergence profile, ensuring correct implant positioning, and conditioning the peri-implant bone accordingly, this approach appears to prevent negative remodeling of the surrounding bone. It ensures the long-term stability of both hard and soft tissue conditions.

## 5. Conclusions

The present study highlights the advantages of fully digitally planned implant restorations in achieving favorable bone morphology outcomes for both immediate (Type 1A) and delayed (Type 4A) procedures. Three-dimensional implant planning, in combination with intraoperative surgical and prosthetic shaping of the supracrestal complex, is crucial to minimize uncontrolled remodeling and establish a stable emergence profile. This approach ensures the maintenance of consistent peri-implant bone levels over time, even with invasive bone shaping. By anticipating the ideal emergence profile and ensuring accurate implant positioning, negative remodeling is prevented, leading to the successful and stable clinical and aesthetic preservation of both the supracrestal complex and surrounding soft tissue.

## Figures and Tables

**Table 1:** Age, gender, implant size, and implant positions are presented. SD represents the standard deviation. The abbreviations for implant localization indicate the position in the jaw: UCI = upper central incisor, ULI = upper lateral incisor, UC = upper canine, UPr = upper premolar, and LPr = lower premolar.

	Group 1 (n = 19)					Group 2 (n = 10)					
Age (years) Mean (SD), Median	48 (±23), 59					50 (±13), 45					
Gender (Female/Male)	5/12					3/6					
Implant Size (Millimeters)	3.7 × 11.5		2			4.2 × 9.5		1			
	3.7 × 14.5		1			4.2 × 11.5		7			
	4.2 × 9.5		1			4.2 × 14.5		1			
	4.2 × 11.5		4			4.7 × 9.5		1			
	4.2 × 13		6								
	4.2 × 14.5		4								
	4.7 × 11.5		1								
Implant Localization	UCI	ULI	UC	UPr	LPr	UCI	ULI	UC	UPr	LPr	
	5	4	3	3	4	2	0	1	5	2	

**Table 2:** The values in the table represent the difference in the highest proximal bone level in millimeters, each referenced to the time of implantation (t0). The values are provided for the total number of implants (overall), as well as for the immediate implantation group (Group 1) and the delayed implantation group (Group 2). The 95%-confidence interval is shown in parentheses.

Δ haBL Mean (mm)	t_0_	t_1_	t_2_	t_3_
Overall	reference	−0.023 (−0.264–0.217)	−0.075 (−0.262–0.113)	−0.269 (−0.547–0.001)
Groupe 1 (Type 1A)		0.034 (−0.381–0.120)	−0.015 (−0.209–0.179)	−0.287 (−0.660–0.883)
Groupe 2 (Type 4A)		−0.131 (−0.381–0.120)	−0.183 (−0.632–0.267)	−0.237 (−0.743–0.270)

**Table 3:** The values shown represent the vertical distance between the Implant Reference Level (IRL) and the first point of Bone-to-Implant Contact (fBIC), measured from crestal to apical. These are the mean values in millimeters for each group (1 and 2) and for the overall dataset (overall). Negative values indicate bone resorption. The 95% confidence interval is shown in parentheses.

Δ fBIC Mean (mm)	t_0_	t_1_	t_2_	t_3_
Overall	Reference	−0.001 (−0.026–0.023)	−0.012 (−0.047–0.008)	−0.013 (−0.041–0.015)
Group 1 (Type 1A)		0.004 (−0.018–0.027)	−0.014 (−0.051–0.023)	0.003 (−0.031–0.036)
Group 2 (Type 4A)		−0.012 (−0.08–0.056)	−0.031 (−0.077–0.016)	−0.042 (−0.094–0.011)

**Table 4:** The values in the table represent the mean measurements in millimeters, illustrating the development of the width of the emergence profile (WEP). The data includes values for both Group 1 and Group 2, as well as for all implants at time points t_1_ (6 months), t_2_ (12 months), and t_3_ (24 months). Each measurement was referenced to t0 (time of implantation). The 95% confidence interval is shown in parentheses.

Δ WEP Mean (mm)	t_0_	t_1_	t_2_	t_3_
Overall	reference	0.027 (−0.045–0.098)	0.154 (0.058–0.250)	0.127 (0.044–0.211)
Group 1 (Type 1A)		0.0003 (−0.088–0.0889	0.089 (−0.004–0.183)	0.103 (−0.014–0.221)
Group 2 (Type 4A)		0.07 (−0.071–0.211)	0.271 (0.052–0.489)	0.170 (0.043–0.296)

## Data Availability

The original contributions presented in this study are included in the article. Further inquiries can be directed to the corresponding author.
